# Plasticity within non-cerebellar pathways rapidly shapes motor performance *in vivo*

**DOI:** 10.1038/ncomms11238

**Published:** 2016-05-09

**Authors:** Diana E. Mitchell, Charles C. Della Santina, Kathleen E. Cullen

**Affiliations:** 1 Department of Physiology, McGill University, 3655 Promenade Sir William Osler, Room 1219, Montreal, Quebec, Canada H3G 1Y6; 2 Department of Otolaryngology—Head & Neck Surgery, Johns Hopkins University School of Medicine, 720 Rutland Avenue, Room 830, Baltimore, Maryland 21205, USA

## Abstract

Although cerebellar mechanisms are vital to maintain accuracy during complex movements and to calibrate simple reflexes, recent *in vitro* studies have called into question the widely held view that synaptic changes within cerebellar pathways exclusively guide alterations in motor performance. Here we investigate the vestibulo-ocular reflex (VOR) circuitry by applying temporally precise activation of vestibular afferents in awake-behaving monkeys to link plasticity at different neural sites with changes in motor performance. Behaviourally relevant activation patterns produce rapid attenuation of direct pathway VOR neurons, but not their nerve input. Changes in the strength of this pathway are sufficient to induce a lasting decrease in the evoked VOR. In addition, indirect brainstem pathways display complementary nearly instantaneous changes, contributing to compensating for the reduced sensitivity of primary VOR neurons. Taken together, our data provide evidence that multiple sites of plasticity within VOR pathways can rapidly shape motor performance *in vivo*.

## Abstract (web summary)

The extent to which non-cerebellar pathways can refine motor performance is debated. Here, the authors demonstrate behaviourally relevant patterns of activation evoke rapid plasticity within direct and indirect vestibulo-ocular reflex pathways *in vivo*, leading to changes in evoked eye movements.

## Graphical abstract

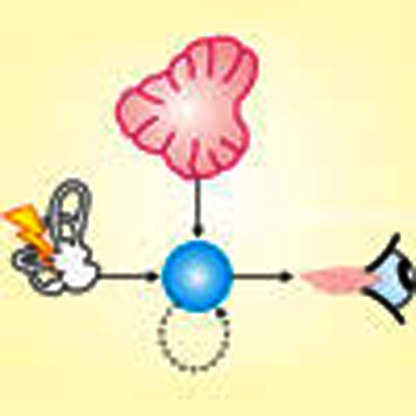

Plasticity within motor pathways plays an essential role in fine-tuning the coordination and accuracy of complex movements as well as in calibrating simple reflex behaviours. To understand the underlying mechanisms, it is necessary to link changes in the patterns of neural activity with specific changes in performance. The simplicity of the vestibulo-ocular reflex (VOR) circuitry and its precise behavioural readout (that is, eye movements) make it an excellent model system for demonstrating how changes in single neuron responses lead to adaptive modification of circuit function and motor behaviour. The direct VOR pathway is mediated by a 3-neuron arc: vestibular afferents send head motion signals to vestibular nuclei neurons, which directly project to extraocular motoneurons (Fig. 1a)^1^. The VOR stabilizes images on the retina by generating compensatory eye movements during head motion^2^. The VOR is capable of remarkable adaptation when there is a mismatch between visual and vestibular stimuli^3^,^4^ as well as following vestibular lesions^5^,^6^,^7^.

The prevailing view is that plasticity within the floccular complex of the cerebellum initially drives VOR adaptation, which in turn triggers longer-term synaptic changes in target neurons within the vestibular nuclei (grey box in Fig. 1a)^8^. It has long been questioned whether plasticity only occurs within this cerebellar-dependent pathway (reviewed in refs 9, 10). Notably, repetitive stimulation of the vestibular nerve decreases the strength of its synapse onto vestibular nuclei neurons *in vitro* (red boxes in Fig. 1a)^11^,^12^. This work suggests that synaptic plasticity can occur within brainstem pathways alongside synaptic changes within the cerebellum, but to date no study has addressed its role in guiding sensory-motor plasticity *in vivo*.

Here we directly tested whether activity-dependent changes in the strength of the first central vestibular synapse contribute to changes in motor performance in awake-behaving monkeys. While activating vestibular afferents within their physiological frequency range of firing and measuring eye movements, we simultaneously recorded from single afferents or their target neurons in the vestibular nuclei. Behaviourally relevant patterns of vestibular nerve activation led to a rapid and substantial decrease in the responses of first-order central neurons that mediate the direct VOR pathway, while the responses of their afferent input remained unchanged. Interestingly, we further found that stimulation caused a coincident decrease in evoked eye movements lasting up to 8 h; however, the relative reduction was significantly less than that of the concurrent decrease in sensitivity of the direct VOR pathway neurons. Accordingly, we tested whether rapid compensatory changes in the strength of indirect inhibitory brainstem pathways balanced the reduced sensitivity of vestibular nerve-vestibular nuclei synapses. In agreement with this prediction, we found that the responses of local inhibitory pathway neurons remained unchanged following stimulation of the vestibular nerve. This was striking, given that a main source of input to these neurons in our unilateral stimulation protocol was the first-order central neurons whose sensitivity did decrease following repetitive nerve activation. Overall, our results suggest that rapid plasticity within indirect VOR pathways contributes to compensating for the reduced efficacy in the direct VOR pathway to ensure a relatively robust behavioural output.

## Results

### Rapid plasticity in the direct VOR pathway

Synaptic plasticity leads to changes in neuronal responses that mediate changes in behavioural performance. To assess whether and how plasticity at the level of the first central vestibular synapse contributes to changes in the VOR, we simultaneously recorded neuronal activity (afferents or central neurons) and eye movements during stimulation of the horizontal semicircular canal in awake-behaving primates. We began by examining changes in the response of neurons that constitute the primary component of the direct VOR pathway and single afferent fibres following behaviourally relevant patterns of vestibular nerve activation and their linkage to changes in the eye movements. We then assessed the effect of vestibular nerve activation on the responses of cerebellar-pontine neurons and those that make up indirect inhibitory vestibular pathways. Finally, we determined whether neural and behavioural responses recover following vestibular nerve activation.

To establish whether the direct VOR pathway shows plasticity to vestibular nerve stimulation, we first quantified the responses of the single neurons that constitute its first central stage of processing (Fig. 2a). Notably, vestibular nerve afferents send vestibular signals to type I position-vestibular-pause (PVP) neurons in the vestibular nuclei, which also receive inputs from other structures, for example, neurons within oculomotor pathways which are vital for the accurate control of gaze (reviewed in ref. 13). Type I PVP neurons in turn project to the extraocular motoneurons on the contralateral side to produce VOR eye movements^2^. The response of the type I PVP neuron illustrated in Fig. 2b was typical of our sample (*N*=15). Specifically, its firing rate increased for contralaterally directed eye positions during spontaneous eye movements (inset, Fig. 2b) and in response to ipsilateral head motion (that is, a type I response; Fig. 2b). In addition, the neuron stopped firing or ‘paused' during quick phases (vertical arrows in Fig. 2b). Quantitative analysis of our type I PVP neuron population revealed that their responses were consistent with previous characterizations of these neurons^2^. Neurons displayed robust responses to head rotation (0.86±0.16 spikes per second per degree per second) that led head velocity (6.6±1.3°). In addition, they increased their activity as a function of contralateral eye position during periods of fixation (1.06±0.2 spikes per second per degree) as well as with contralateral eye position and velocity during smooth pursuit eye movements (1.47±0.28 spikes per second per degree and 0.4±0.09 spikes per second per degree per second, respectively).

**Figure 1:**
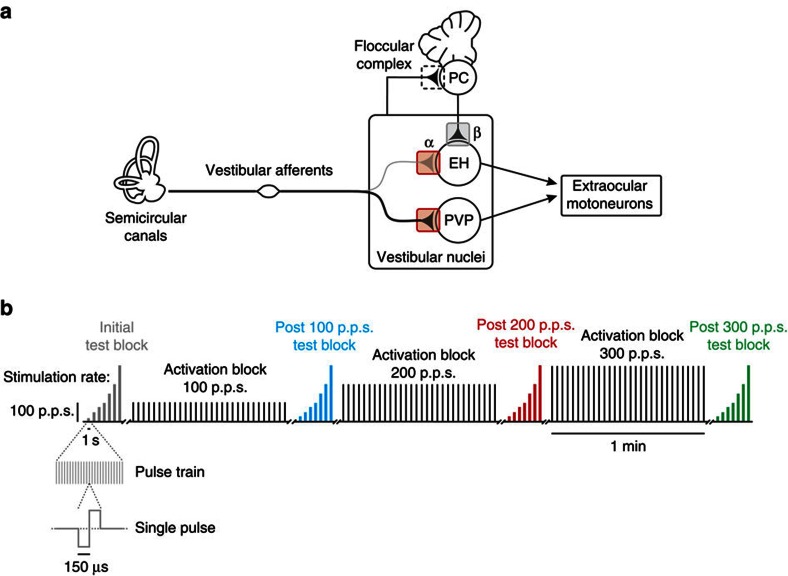
Diagram of VOR circuitry. (**a**) Vestibular afferents project to PVP neurons, which in turn send projections to extraocular motoneurons. EH neurons receive direct input from the vestibular nerve and from floccular Purkinje cells, and they project to extraocular motoneurons. (**b**) Schematic of electrical stimulation protocols. Each rectangle represents a pulse train where the height is the rate and the width is the duration. During test blocks, each pulse train lasted 1 s and rates of 25–300 p.p.s. were used. During activation blocks, 30 pulse trains, each lasting 500 ms, were delivered over the course of 1 min at 100, 200 and 300 p.p.s.

**Figure 2:**
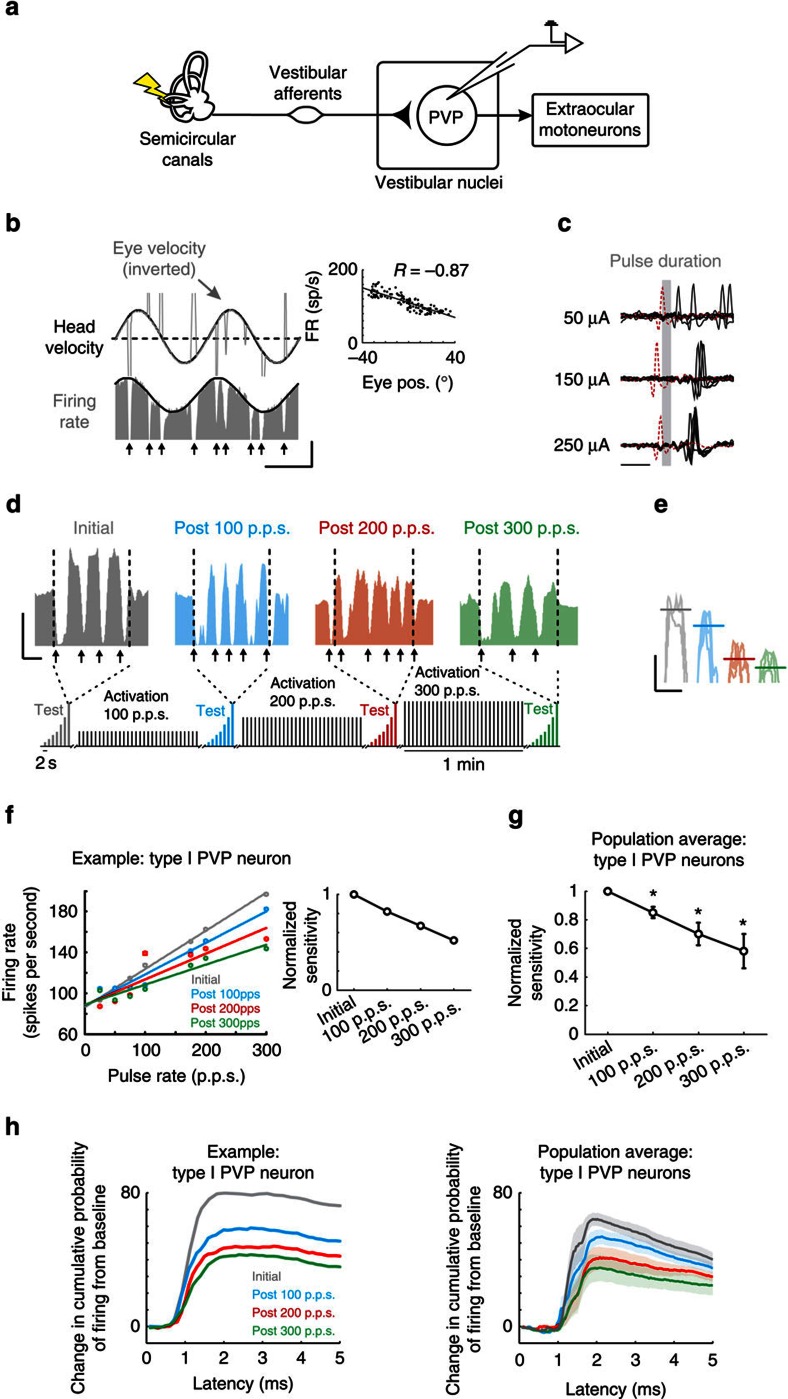
Rapid plasticity within direct VOR pathways. (**a**) Diagram of direct VOR circuitry. (**b**) Firing rate of example type I PVP neuron during VOR. Note eye velocity is inverted and the ipsilateral direction relative to side of recording is upwards. Arrows highlight when saccades occurred. Inset shows example neuron's eye position sensitivity. Calibrations bars, 40 ° per s, 100 spikes per s, 1 s. (**c**) Same example neuron's response to single pulses of increasing current amplitude. Red traces indicate trials during which the neuron failed to fire an action potential because the pulse occurred during its refractory period. Calibration bar, 1 ms. (**d**) Firing rate before (grey) and after activation of the vestibular nerve with rates of 100 (blue), 200 (red) and 300 p.p.s. (green) in response to test stimuli delivered at 300 p.p.s. Note that following activation, the response of the neuron decreased. Inset shows schematic of electrical stimulation protocols. Arrows show when saccades occurred. Calibrations bars, 100 spikes per s, 0.25 s (**e**) Periods of firing rate during the compensatory portion of the eye movement for the example neuron are superimposed to show the decreased response after activation of the vestibular nerve. Calibrations bars, 50 spikes per s, 0.25 s. (**f**) Plot of firing rate as a function of pulse rate for example neuron before and after activation of the vestibular nerve. Inset shows normalized sensitivity (slope of firing rate versus pulse rate) for example neuron. (**g**) Population average of normalized sensitivity for type I PVP neurons (*n*=15). *P<0.05 (Student's *t*-test). (**h**) Probability of firing as a function of latency following individual pulses for example neuron (left) and population averages (right) before and after activation of vestibular nerve. Error bars and shaded area represent ±s.e.m.

Figure 2c shows the response of the same example type I PVP neuron to electrical activation of the ipsilateral vestibular nerve in complete darkness. Notably, we evoked action potentials at monosynaptic latencies (0.7–1.3 ms)^14^,^15^, confirming a direct projection from vestibular afferents (Fig. 2c). Having established this connectivity, we next addressed our main question, namely: does repeated activation of the vestibular nerve induce plasticity at the first central stage of the direct VOR pathway (red boxes in Fig. 1a)? If this is the case, then we would expect that, over time, type I PVP neurons should show a change in their response to repeated activation at a monosynaptic latency. Figure 2d–h verify this prediction. To quantify the change in neuronal response, we delivered a short test stimulus that consisted of pulse trains ranging from 25 to 300 pulses per second (p.p.s.) (that is, test block). While the example neuron showed a robust increase in firing rate with increasing pulse rate for the initial test block (for example, 194±1.2 versus 106±1.1 spikes per second for 300 p.p.s. stimulation versus resting rate, respectively; grey panel in Fig. 2d,e), its response to the same test stimuli decreased following each activation block. For example, following 1 min of stimulation at 100 p.p.s., the example neuron's firing rate response to this same test stimulation decreased to 172±1.1 spikes per second (blue panel in Fig. 2d,e). Moreover, after subsequent activation blocks of 200 and 300 p.p.s., the neuron displayed an even more striking response reduction (129±0.7 and 117±1.0 spikes per second, respectively; red and green panels in Fig. 2d,e).

Figure 2f quantifies the example neuron's response to each pulse rate within our test stimulus. Note that our analysis was restricted to periods of compensatory VOR eye movement, since type I PVP neurons are silenced by the saccadic burst generator during saccades, including the quick phases of nystagmus (reviewed in ref. 13; see arrows in Fig. 2d). The grey line represents the linear relationship between stimulation rate and firing rate before the activation blocks. Although the neuron's firing rate still increased linearly with pulse rate after vestibular nerve activation with 100 p.p.s., it was less sensitive (blue data points in Fig. 2f). This decrease in sensitivity became even more pronounced following activation at 200 and 300 p.p.s. (red and green data points in Fig. 2f). To quantify the decrease in neuronal sensitivity, we computed the slope of the linear regression between firing rate and pulse rate for each of the four test stimuli (inset in Fig. 2f). Similar results were found across our population of recorded neurons (Fig. 2g). Notably, neuronal response sensitivity was significantly decreased following each activation block relative to initial values (*P*<0.05). Furthermore, this finding was unaffected when we constrained our analysis to the first 10 ms and first 100 ms following pulse train onset (Supplementary Fig. 1a), indicating that response attenuation occurred rapidly and remained constant. In contrast, there was no change in the resting rate or resting discharge regularity (see Methods) following each activation block for either type I PVP neurons, or any other of the neuron classes considered below (*P*<0.05).

To more precisely establish the time course of the observed attenuation of type I PVP neurons, we next computed the cumulative probability of evoking a spike as a function of latency following individual pulses (see Methods). We found that responses remained monosynaptically phase-locked to the individual pulses, and importantly were attenuated immediately, at monosynaptic latencies (Fig. 2h and Supplementary Fig. 1b). Moreover, the level of attenuation increased on this same monosynaptic time scale with increasing pulse rate. Together, these results suggest that increased activation of the vestibular afferents induces plasticity at the afferent-PVP synapse within the direct VOR pathway.

### Vestibular afferent responses to stimulation remain robust

**Figure 3:**
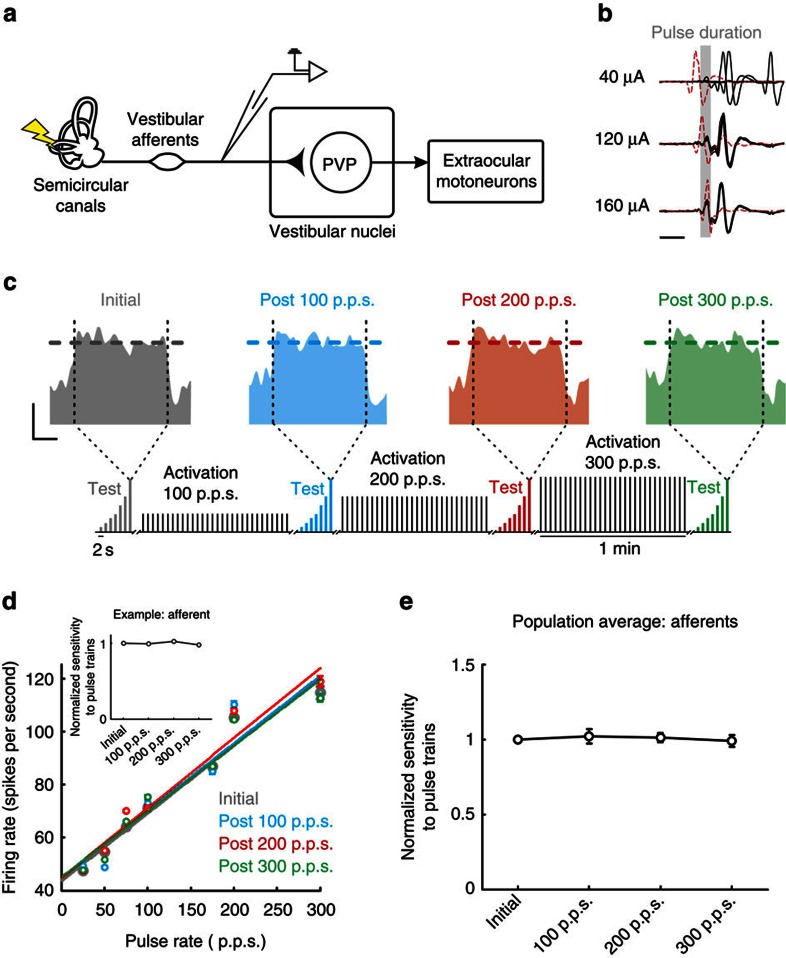
Responses of vestibular afferents remain constant following activation. (**a**) Diagram of direct VOR circuitry. (**b**) Potentials recorded from single afferent fibres in response to single pulses delivered at different current amplitudes. Red traces indicate trials during which the neuron failed to fire an action potential because the pulse occurred during its refractory period. Calibration bar, 1 ms. (**c**) Firing rate before (grey) and after activation of the vestibular nerve with rates of 100 (blue), 200 (red) and 300 p.p.s. (green) in response to test stimuli delivered at 300 p.p.s. Note that the response of the neuron remains constant. Inset shows schematic of electrical stimulation protocols. Calibrations bars, 50 spikes per s, 0.25 s. (**d**) Plot of firing rate as a function of pulse rate for example neuron before and after activation of the vestibular nerve. Inset shows normalized sensitivity (slope of firing rate versus pulse rate) for example afferent. (**e**) Population average of normalized sensitivity for vestibular afferents (*n*=7). Error bars represent ±s.e.m.

Electrical stimulation of the labyrinth is thought to predominantly act at the spike initiator zone of primary vestibular afferents^16^, yet stimulation can also directly alter the transmembrane potential of the vestibular hair cells^17^. Thus, it is possible that our stimulation protocol caused changes at the synapse between hair cells and vestibular afferents that contributed to the decreased type I PVP neuron response (for example, a reduction in neurotransmitter release and/or excitotoxicity due to excessive glutamate release). If this were the case, then we would expect that the response of vestibular afferents would also decrease following repeated activation. However, this is not what we observed when we recorded from single vestibular afferent fibres (*N*=7; Fig. 3a) before and after activation. Figure 3b shows the response of an example afferent fibre to electrical activation of the ipsilateral vestibular nerve. Notably, action potentials were evoked almost instantaneously (<500 μs). Moreover, in contrast to what we observed for type I PVP neurons, we found that the response of single afferent fibres to the test stimulus remained constant throughout the experimental trial (Fig. 3c,d). The example cell was typical in that it showed a robust increase in firing rate with increasing pulse rate for the initial test block (119±2.3 versus 43±0.6 spikes per second for 300 p.p.s. stimulation versus resting rate, respectively; grey panel in Fig. 3c). Furthermore, following 1 min of stimulation during the activation blocks at 100, 200 and then 300 p.p.s., the example neuron's firing rate remained constant (122±1.6, 124±2.2 and 119±1.5 spikes per second, respectively, for 300 p.p.s. test stimulation; blue, red and green firing rates in Fig. 3c).

Figure 3d quantifies the example cell's response as a function of the pulse rates within our test stimulus. The grey line represents the linear relationship between stimulation rate and firing rate before the activation blocks. Notably, following each activation block, the relationship between the example neuron's firing rate and stimulation rate remained constant (compare grey, blue, red and green data points in Fig. 3d). To quantify the neuronal sensitivity, we computed the slope of the linear regression between firing rate and pulse rate for each of the four test stimuli (inset in Fig. 3d). These results were consistent for the population of vestibular afferents from which we recorded; the neural sensitivities were not significantly different following any of the activation blocks (*P*>0.05; Fig. 3e). Therefore, repeated activation of the vestibular periphery does not alter the sensitivity of single afferent fibres over the duration of our experiment.

### Neuronal plasticity alters behavioural performance

**Figure 4:**
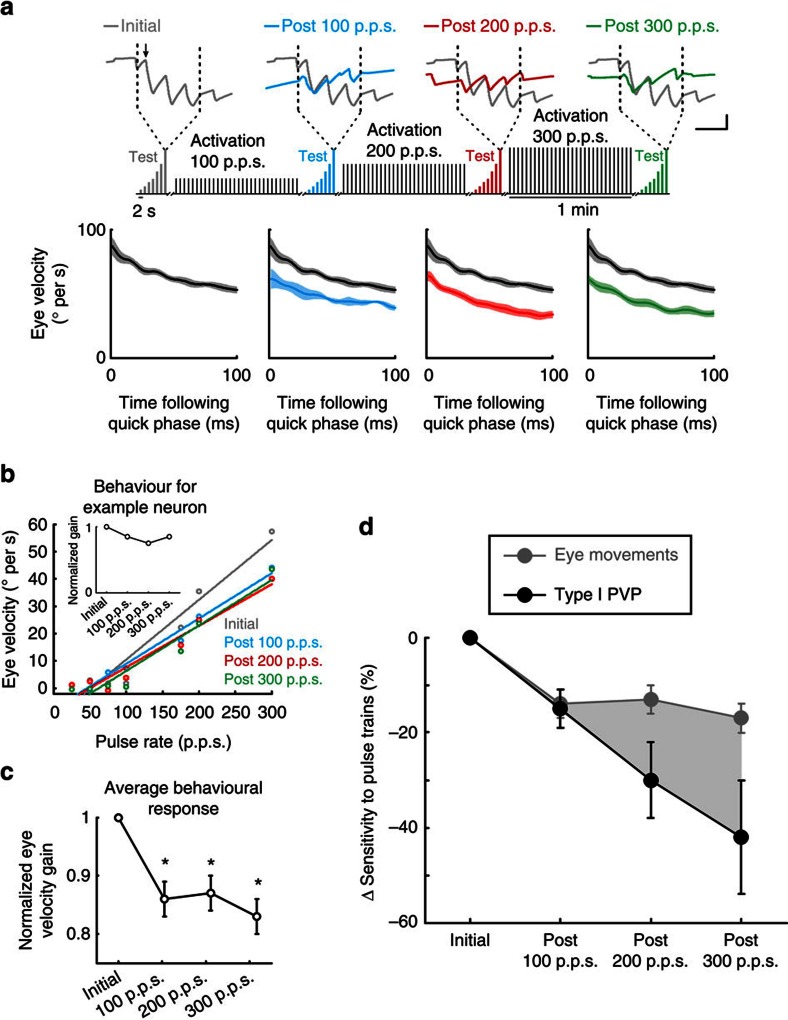
Eye movement responses decrease following activation of the vestibular nerve. (**a**) Top panel: eye position traces during test stimuli at 300 p.p.s. before and after activation of the vestibular nerve. Inset shows schematic of electrical stimulation protocols. Arrow highlights example quick phase. Bottom panels: averaged eye velocity over the 100 ms following each saccade that occurred throughout the 1 s of stimulation at 300 p.p.s. Calibrations bars, 10 °, 0.5 s. (**b**) Average eye velocity (restricted to periods of compensatory VOR) as a function of pulse rate for the corresponding eye movements recorded with the example neuron in Fig. 2. Inset shows normalized gain (slope of eye velocity versus pulse rate). (**c**) Average normalized gain across all trials (*n*=61). *P<0.05 (Student's *t*-test). (**d**) Comparison between decrease in sensitivity for type I PVP and eye movement responses. Error bars and shaded area represent ±s.e.m.

The results presented so far demonstrate that repeated activation of the vestibular nerve reduces the efficacy of the direct VOR pathway by altering how type I PVP neurons integrate their afferent input. Given that type I PVP neurons project directly to extraocular motoneurons (Fig. 2a), and principally constitute the intermediate stage of the direct VOR pathway, it follows that the evoked VOR eye movements should show a parallel decrease. To test this proposal, we quantified the eye movements that were measured simultaneously during the neuronal recordings described above, again restricting our analysis to periods of compensatory VOR. Initially, stimulation at each of the pulse rates in the test stimulus produced increasingly robust compensatory vestibular nystagmus (that is, with a slow phase directed contralaterally away from the side of the stimulated nerve; Fig. 4a, grey trace in top panel) with an average velocity of ∼50° per second during 300 p.p.s. stimulation (Fig. 4a, grey trace in bottom panel). However, following the first activation block (that is, stimulation at 100 p.p.s. for 1 min), the behavioural response was diminished (blue versus grey traces in bottom panel of Fig. 4a). Similarly, the eye movements evoked by the test stimulus were reduced following the second and third activation blocks (grey versus red and green traces in bottom panel of Fig. 4a). Note, the attenuation remained relatively constant following the additional activation of the nerve, and no post-stimulation nystagmus was observed.

To quantify the observed change in behavioural performance, we computed the average of the eye velocity, over epochs of compensatory VOR, evoked by each of our seven test stimuli (that is, range 25–300 p.p.s., Fig. 4b). Test stimuli evoked robust responses that were linearly related to pulse rate before the application of our activation paradigm (Fig. 4b, grey trace). However, responses were attenuated following activation blocks (grey versus blue, red and green traces). To assess the change in sensitivity to nerve activation, we quantified the slope of these curves before and after activation of the vestibular nerve, and found that, on average, the eye movement sensitivity was significantly decreased following activation (*P*<0.05; Fig. 4c), validating our hypothesis that vestibular nerve stimulation does in fact cause a decrease in eye movement responses. Comparable results were also obtained when we restricted our analysis to the first 10 ms and first 100 ms following pulse train onset (Supplementary Fig. 1c), indicating that behavioural response attenuation occurred rapidly and remained constant.

Having established that both type I PVP neurons and the VOR eye movements show rapid reduction in sensitivity following repetitive vestibular nerve stimulation, we next directly compared the relative magnitude and time course of these changes. The normalized changes in sensitivity of type I PVP neurons and the eye movements are superimposed in Fig. 4d. While both sensitivities decrease following activation of the vestibular nerve, it is clear that neuronal responses subsequently decrease by a greater amount. For example, following the third activation block (that is, stimulation at 300 p.p.s. for 1 min), the average neuronal sensitivity decreased by 42% while the behavioural sensitivity dropped by only 17% (compare black and grey symbols).

### Rapid complementary changes in inhibitory brainstem pathways

In addition to receiving direct input from ipsilateral vestibular afferents, central neurons in the vestibular nuclei receive indirect contralateral input through the vestibular commissural pathways^18^. This input is commonly thought to help maintain balanced activity at rest^19^, as well as increase the response sensitivity to rotational head motion^20^,^21^,^22^. Furthermore, long-term changes in the commissural connections between the vestibular nuclei play a vital role in the compensation observed following unilateral vestibular loss^23^,^24^. Vestibular nuclei neurons also receive input from ipsilateral inhibitory neurons, which are thought to serve a similar role in pathway calibration and compensation^25^. If changes in these local inhibitory pathways compensate for the reduced sensitivity of the type I PVP neurons following repetitive vestibular nerve stimulation, then we should see evidence for complementary changes on the same rapid time scale.

**Figure 5:**
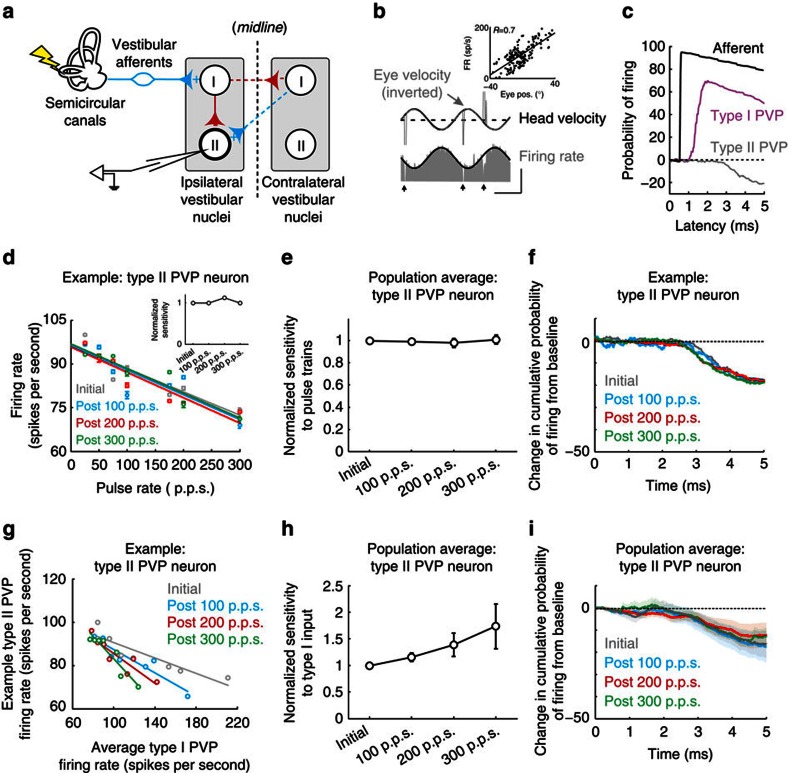
Rapid complementary changes in indirect inhibitory brainstem pathways. (**a**) Diagram of local inhibitory pathway within the vestibular nuclei. (**b**) Firing rate of example type II PVP neuron during VOR. Arrows highlight when saccades occurred. Inset shows example neuron's eye position sensitivity. Calibrations bars, 100 ° per s, 100 spikes per s, 1 s. (**c**) Probability of firing as a function of latency from onset of individual pulses for vestibular afferent, as well as for type I and II PVP neurons. (**d**) Plot of firing rate as a function of pulse rate for example neuron before and after activation of the vestibular nerve. Inset shows normalized sensitivity (slope of firing rate versus pulse rate) for example type II PVP neuron. (**e**) Population average of normalized sensitivity for type II PVP neurons (*n*=10). (**f**) Probability of firing as a function of latency following individual pulses for example neuron before and after activation of vestibular nerve. (**g**) Input–output relationship between type I and type II PVP neurons (that is, type II PVP example neuron firing rate as a function of average type I PVP neuron firing rate) before and after activation of the vestibular nerve. (**h**) Population average of normalized sensitivity to type I input for type II PVP neurons (*n*=10). (**i**) Population averaged probability of firing as a function of latency following individual pulses before and after activation of vestibular nerve. Error bars and shaded area represent ±s.e.m.

We therefore next investigated the response of type II PVP neurons ipsilateral to the side of stimulation. Type II PVP neurons are modulated via local inhibitory pathways, with the most direct being uncrossed disynaptic^11^,^25^ (red solid line in Fig. 5a) and crossed trisynaptic inhibition^14^,^18^ (red dashed line in Fig. 5a). In addition, as is shown for the example neuron, their responses are complementary to those of type I PVP neurons; firing rates increased for ipsilaterally directed eye positions during spontaneous eye movements (inset, Fig. 5b) and in response to contralateral head motion (that is, a type II response; Fig. 5b). Figure 5c compares the probability of firing an action potential in response to electrical activation of the ipsilateral vestibular nerve for the example afferent, as well as for example type I and type II PVP neurons. Note that while we consistently evoked action potentials almost instantaneously for afferents and at monosynaptic latencies for type I PVPs, the same stimulation actually reduced the probability of type II PVP firing and the response latency was significantly longer (2.5±0.2 ms), consistent with the polysynaptic inhibitory pathway shown in Fig. 5a.

Having established the indirect connection between these neurons and the ipsilateral vestibular nerve, we next addressed our second main question, namely: does repeated activation of the vestibular nerve rapidly induce compensatory changes through this indirect pathway? Figure 5d shows the same example neuron's sensitivity to our test stimulus before and following each of the three activation blocks, in which stimulation was applied at rates of 100, 200 or 300 p.p.s., respectively. During our initial test block, this neuron was typical in that its firing rate decreased linearly with increasing pulse rates (grey data points in Fig. 5d). Remarkably, however, we found no change in this relationship following any of the three subsequent activation blocks (blue, red and green data points in Fig. 5d). Instead, the inhibitory relationship between pulse rate and firing rate remained constant (*P*>0.05, *N*=10). Moreover, as done above in our analysis of type I PVP neurons and vestibular afferents, we quantified the slope of this relationship to compute the neuron's sensitivity to the test stimuli (inset in Fig. 5d). As expected, type II PVP neurons' sensitivity did not change following repetitive nerve activation (Fig. 5e).

To establish whether this result held over a more precise time scale time, we next computed the probability of evoking a spike as a function of latency following individual pulses (Fig. 5f–i, see Methods). We found that the probability of a spike occurring following stimulation remained constant after vestibular nerve activation with each pulse rate (Supplementary Fig. 2a). In addition, there was no change in latency (Supplementary Fig. 2b); the probability of firing decreased with a latency consistent with the disynaptic/trisynaptic connectivity of type II PVP neurons to the nerve (Fig. 5f–i).

The above finding is rather surprising, particularly in light of the fact that type I PVP neurons, whose responses do decrease following repetitive nerve activation (Fig. 2g), provided the main source of driven input to type II PVP neurons in our unilateral stimulation protocol (Fig. 5a). To better understand the implications of this finding, we next characterized the input–output relationship of type II neurons by plotting their output firing rates as a function of the activation of their type I PVP neuron input rather than test stimulation pulse rate (Fig. 5g). Note that the slope of the input–output relationship between type I and type II PVP neurons effectively serves as an estimate of the efficacy of local inhibitory pathways within the vestibular nuclei. If there were no change in the efficacy of these pathways, then type II PVP responses should follow that of their input (that is, type I PVP neurons), and thus become less inhibited following vestibular nerve activation. However, we found that the sensitivity of type II neurons actually increased (that is, became more inhibitory) relative to its input following activation of the vestibular nerve (Fig. 5h). Thus, taken together, these results are consistent with the proposal that rapid changes in local inhibitory pathways compensate for the decreased response of the type I PVP neurons, thereby allowing the VOR circuitry to maintain a relatively robust behavioural output.

Finally, we completed a comparable analysis of another distinct physiological subclass of neurons in the vestibular nuclei, termed eye-head (EH) neurons. These neurons receive strong projections from the floccular lobe of the vestibular cerebellum in addition to the ipsilateral vestibular nerve, and in turn project to extraocular motoneurons (Supplementary Fig. 3a)^2^. EH neurons were identified based on their characteristic responses to gaze and head movements in the same direction during horizontal smooth pursuit eye movements and cancellation of the VOR, respectively (Supplementary Fig. 3b). The input from this cerebellar-pontine pathway is thought to complement the direct VOR pathway to keep the performance of the VOR calibrated over time. Accordingly, we asked whether rapid changes within cerebellar-pontine pathways also compensate for the reduced sensitivity of the type I PVP neurons following repetitive vestibular nerve activation. If this is the case, then we should see evidence of complementary changes in the responses of EH neurons on the same rapid time scale as seen above for type I PVP neurons (that is, Figs 2 and 5). Instead, we found that the change in sensitivity of EH neurons was much less following repetitive vestibular nerve activation compared with type I PVP neurons (Supplementary Fig. 3c). Specifically, the sensitivity of contralateral EH cells (*N*=14) only showed a significant decrease following activation of the vestibular nerve at 300 p.p.s., while ipsilateral EH cells (*N*=12) did not show any significant decrease in their response sensitivity. Moreover, both the latency and magnitude of the response to individual pulses remained constant following vestibular nerve activation (Supplementary Fig. 4). Thus, our results show that repeated activation of the vestibular nerve does not alter the sensitivity of EH cells, indicating that floccular projections do not make a major contribution to the rapid plasticity observed in our experiments (see Discussion). Taken together, the above results suggest that rapid changes within the local inhibitory brainstem but not cerebellar pathways compensate for the decreased response of the type I PVP neurons to ensure a relatively robust VOR performance.

### Recovery of neuronal and VOR responses

**Figure 6:**
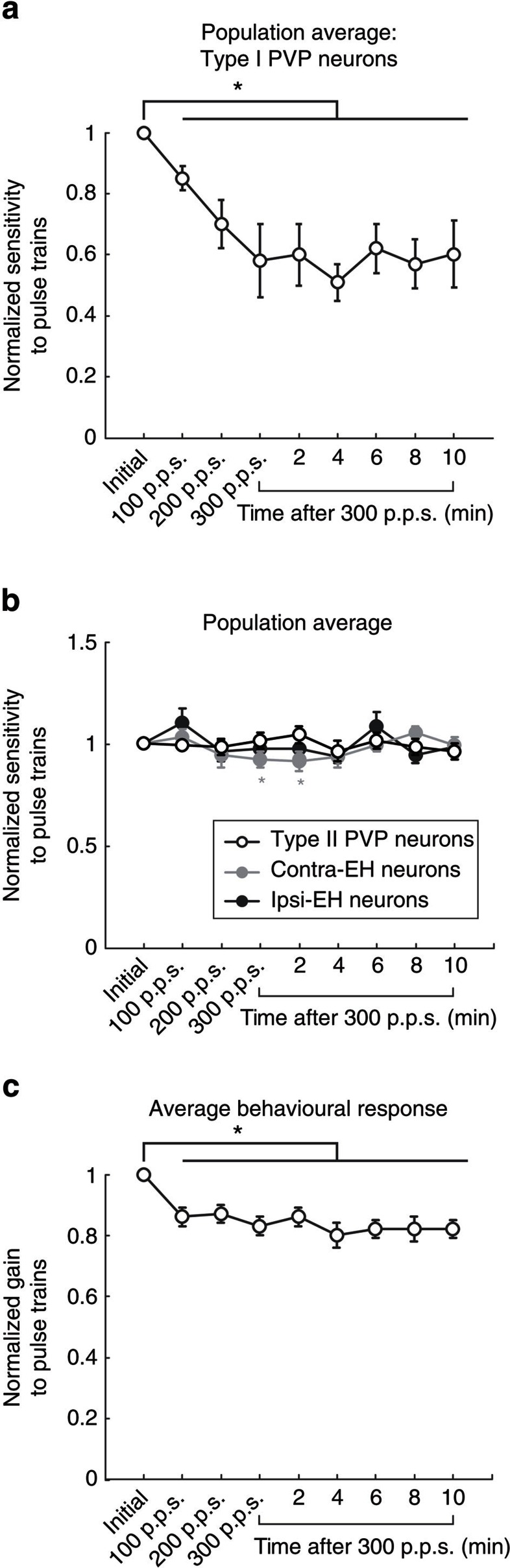
Recovery of neuronal and behavioural responses following activation. (**a**,**b**) Normalized sensitivity of type I PVP neurons (*n*=15) as well as type II PVP neurons (*n*=10), contra EH cells (*n*=14) and ipsi EH cells (*n*=12) (**b**) over a 10-min period following activation of the vestibular nerve. (**c**) Normalized eye velocity gain over a 10-min period following activation of the vestibular nerve. Error bars represent ±s.e.m.

The results presented above show that repeated activation of the vestibular nerve rapidly depresses the sensitivity of neurons at the first central stage of the direct VOR pathway. While these results are consistent with previous *in vitro* work showing long-term depression (LTD) at the vestibular nerve-vestibular nuclei synapse^11^,^12^, it is also important to establish whether the plasticity observed in our *in vivo* experiments persisted over a more extended period. Thus, to establish the time course of the synaptic depression, we continued to quantify the sensitivity of neurons using test blocks applied every 2 min for up to 10 min following the last activation block. We found that while the sensitivities of type I PVP neurons were maximally attenuated 4 min following the final activation block (Fig. 6a), after 10 min neural responses were still significantly attenuated (40%, *P*<0.05) relative to initial values. In contrast, the responses of type II PVP neurons as well as those of ipsi- and contra-EH cells remained relatively constant over this time period (Fig. 6b). The only significant change measured by the end of this interval was a decrease in the neural sensitivity of contra-EH cells immediately after and 2 min following the most powerful of the test stimuli of 300 p.p.s. (*P*<0.05). Similarly, behavioural responses remained significantly attenuated relative to initial values over this same period (Fig. 6c).

**Figure 7:**
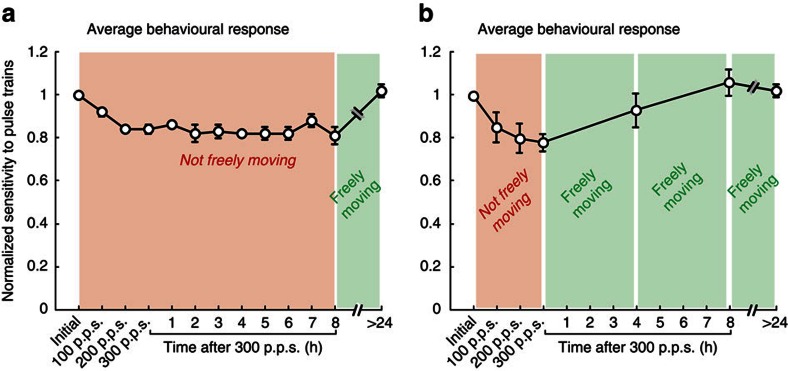
Behavioural recovery following vestibular nerve activation. (**a**) Normalized eye velocity gain over an 8-h period following activation of the vestibular nerve when the animal remained stationary. (**b**) Normalized eye velocity gain over an 8-h period following activation of the vestibular nerve when the animal was returned to its home cage in between testing sessions. Error bars represent ±s.e.m.

So far, our results show that the dynamics of the plasticity observed in our *in vivo* experiments are consistent with those in prior *in vitro* studies that characterized LTD at the vestibular nerve-central neuron synapse^11^,^12^, and that, in turn, this plasticity triggers a lasting reduction in the evoked eye movement. Given that both neural and behavioural responses typically remained attenuated, we next addressed (1) whether the time course of plasticity is significantly longer and (2) what conditions might facilitate the return of synaptic efficacy to baseline levels. Prior *in vitro* studies in other pathways have reported LTD lasting for hours to days^26^,^27^. To determine whether the effects of our vestibular nerve activation persist on this longer time scale, we continued to apply our test stimuli at hourly intervals for a period of 8 h following activation of the vestibular nerve and quantified the evoked eye movements. Notably, the evoked VOR eye movements remained significantly attenuated throughout this 8 h period when the animal remained stationary in the dark between tests (Fig. 7a). In contrast, the behavioural responses recovered within ∼4 h (Fig. 7b) if the animal was returned to its home cage following each testing session. Thus, we found that the time course of plasticity can last for up to 8 h, but that stimulation of the pathway with ‘natural motion' facilitates recovery of the afferent to central neuron synapse (that is, return to baseline), restoring the VOR pathway efficacy.

## Discussion

The results from this study provide new insight into the mechanisms that mediate changes in VOR motor performance. Behaviourally relevant rates of vestibular nerve activation caused a decrease in the monosynaptic response of direct VOR neurons, yielding a lasting reduction in evoked eye movements. In contrast, we found that the response of single vestibular afferent fibres remained unchanged when recorded under similar conditions. Together these results establish that vestibular nerve activation induces rapid (<1 min) plasticity at the vestibular afferent to central neuron synapse in awake-behaving primates. Interestingly, the relative decrease in sensitivity of direct VOR neurons was considerably more than the observed change in motor performance. To understand this apparent discrepancy, we recorded from neurons within the local inhibitory pathways of the vestibular nuclei, and found evidence for an enhancement of the relative weight of this indirect pathway on the same rapid timescale. Thus, our findings reveal a novel mechanism for plasticity in which remarkably rapid changes in local inhibitory inputs offset the decreased efficacy of direct sensory-motor pathways to enhance behavioural performance.

The time course of the plasticity observed in our *in vivo* experiments was consistent with that reported in prior *in vitro* studies of LTD at the vestibular nerve-central neuron synapse^11^,^12^,^28^, which have described effects lasting from 30 min up to 2 h. Investigations of synapses in other sensory-motor circuits have demonstrated that LTD can persist over even longer time scales, ranging from hours to days^26^,^27^,^29^. Here our experiments further revealed a marked and sustained reduction in synaptic efficacy, which in turn produced a change in motor performance that endured for up to 8 h. Importantly, behavioural recovery was appreciably faster (<4 h) when monkeys were allowed to move freely between test blocks than when they remained relatively stationary. This suggests that the strength of the depressed afferent-central synapse did not passively return to baseline, but instead its recovery was mediated by an active process. We speculate that the sensory stimulation induced by natural movement triggers a form of homeostatic plasticity in which contralateral vestibular input, as well as inputs from other modalities, help restore network activity to a set point level in response to changes in the efficacy of vestibular input. Indeed, extra-vestibular inputs including neck proprioceptive and motor efference copy signals can substitute for vestibular signals at the level of single neurons within the direct VOR pathway following peripheral vestibular loss^30^,^31^. This finding is likely to have general significance, since similar changes in network activity have been shown to produce compensatory long-term changes in the excitability of synapses in other sensory systems including auditory^32^ and visual^33^ cortex.

Another fundamental difference between the vestibular input experienced in normal everyday life and our stimulation experiments is that, in the latter case, afferent spiking activity is synchronized since each pulse of the applied stimulation train reliably (and nearly simultaneously) produces an action potential in individual vestibular afferents (Fig. 3b). In contrast, during applied head movements, vestibular afferents show little to no synchrony^34^,^35^. This difference provides a possible explanation for our finding that while the decrease in behavioural responses can last for up to 8 h when there is minimal natural vestibular input, subsequent asynchronous stimulation of the pathway with ‘natural motion' facilitates a return to baseline (that is, Fig. 7a versus Fig. 7b). Interestingly, we further found that the sensitivity of cerebellar-pontine neurons did not change in response to nerve activation—even though these neurons receive some direct ipsilateral vestibular nerve input in addition to projections from the flocculus^2^. We speculate that this is in part because the indirect input from the cerebellum (β versus α in Fig. 1a) helps to desynchronize the overall input to these neurons, in contrast to type I PVP neurons that primarily receive direct input from the vestibular nerve.

Comparison of type II and type I PVP neurons showed that local inhibitory pathways undergo rapid plasticity that compensates for the decreased response of type I PVP cells. Specifically, the lack of change in the relationship between nerve activation and type II neuron sensitivity suggests an increase in the sensitivity of these neurons to their input from type I vestibular nuclei neurons. A similar interplay between plasticity mechanisms has been reported in other neural circuits. Chronic electrical stimulation in organotypic slices of primary auditory and somatosensory cortex show an increase in evoked polysynaptic activity despite decreasing evoked monosynaptic responses^36^. Strengthening polysynaptic connections to offset decreased monosynaptic weights within the direct VOR circuitry could be beneficial for motor performance. Furthermore, prior studies in cerebellum, with protocols similar to those used in the present study, reported an enhancement in the synaptic strength of inhibitory pathways following stimulation^37^. We found evidence that supports such parallel changes in the vestibular nuclei, where the depression of the monosynaptic excitatory VOR pathway neurons ipsilateral to the stimulated nerve is counterbalanced by complementary changes in these polysynaptic inhibitory pathways. Notably, these pathways include local networks within the ipsilateral vestibular nucleus^11^,^25^ as well as commissural pathways^14^,^18^.

It should be emphasized that to date *in vivo* studies typically characterize VOR pathways based on neuronal firing rate responses during oculomotor behaviours, while *in vitro* studies identify cell types on the basis of their projections and genetic makeup, making it difficult to bridge these two bodies of work. However, recent progress has been made towards elucidating the circuitry of vestibulo-cerebellar pathways as well as local circuit networks. For example, type I PVP neurons most likely comprise glutamatergic neurons that project to extraocular motoneurons as described by McElvain *et al*.^11^. In addition, distinct subtypes of floccular-target neurons have been identified based on their degree of cerebellar innervation^38^. Future work aimed at determining how these neural populations compare with the physiologically identified subclasses presented here will have important implications for determining the cellular mechanisms underlying the rapid compensation observed in the present study.

Climbing fibre input to the cerebellum is considered to play a major role in guiding cerebellar LTD to induce a change in VOR motor performance^39^,^40^,^41^. Our results, however, provide evidence that synaptic changes in non-cerebellar pathways can mediate plasticity *in vivo*. Specifically, we found that changes in VOR efficacy occur as a result of depression of the direct VOR pathway. Interestingly, the results of other recent studies have also called into question the view that climbing fibre input and/or plasticity within cerebellar pathways is required^42^,^43^. In addition, motor-learning deficits are not complete in transgenic mice with impaired cerebellar LTD^44^,^45^,^46^,^47^. Furthermore, rapid homeostatic plasticity within the direct VOR pathway can improve motor performance following the loss of vestibular nerve input^30^,^31^. Taken together, the results of the present and previous studies provide support for the notion that there are multiple sites of plasticity that shape motor performance even in simple pathways such as the VOR.

Patients with loss of peripheral vestibular function suffer from debilitating symptoms including postural and gaze instability. Vestibular prosthetic devices offer the promise of recovered functionality to these individuals by activating vestibular pathways through electrical stimulation of the vestibular nerve. Notably, our findings have implications for the successful implementation such devices since existing vestibular prosthetic models stimulate the vestibular nerve at rates comparable to those applied in this study^48^,^49^,^50^,^51^,^52^. Based on our results, we predict that stimulation protocols presently used would induce plasticity at the afferent to first-order central neuron synapse, which in turn would result in decreased behavioural performance. Indeed, to date, vestibular prosthetic stimulation typically produces appropriately directed reflexive eye and head movements^50^,^51^,^52^, but these responses are reduced relative to those produced by natural stimulation. Interestingly, the brain can learn to integrate artificial sensory signals to accomplish certain tasks or functions (for example, somatosensory system^53^). Further work will be required to develop stimulation strategies that take advantage of how the brain processes such artificial sensory inputs, and will likely improve the behavioural performance of the vestibular prosthesis.

Our findings establish a link between VOR motor performance and plasticity within different sites of the VOR circuit *in vivo*. Our results suggest that indirect inhibitory brainstem pathways undergo rapid plasticity to compensate for the decreased weight of direct VOR pathways ultimately allowing the maintenance of a relatively robust behavioural output. Thus, this work provides a foundation for understanding how coordinated plasticity within specific sites of a simple sensory-motor pathway guides motor learning to ensure robust performance. Finally, our work also has implications for design of stimulation protocols used in vestibular prostheses to treat patients disabled by loss of sensory input from both labyrinths.

## Methods

### Surgical procedures

Three male rhesus monkeys (aged 4, 5 and 7 years old) were prepared for chronic behavioural experiments. All procedures were approved by both the McGill University Animal Care Committee and the Johns Hopkins Animal Care and Use Committee, in addition to following the guidelines of the Canadian Council on Animal Care and the National Institutes of Health.

Surgeries in preparation for neuronal recordings and VOR measurement were performed as described by Sylvestre and Cullen^54^. Anaesthesia was achieved by the use of isoflurane gas (2–3% initially) and maintained for the duration of the procedure (0.8–1.5%). A stainless steel post (used to immobilize the monkey's head) was attached to the skull of the animal using dental acrylic and stainless steel screws. Two stainless steel recording chambers were attached to the implant, each positioned to provide access to the medial vestibular nucleus or the left vestibular nerve where it emerges from the internal auditory meatus. A 16–17-mm eye coil, consisting of three loops of Teflon-coated stainless steel wire, was sutured to the sclera and beneath the conjunctiva of the right eye.

Implantation of stimulating electrode array was performed similarly to procedures described by Dai *et al*.^55^. An electrode array was implanted into the left labyrinth via a transmastoid approach. Specifically, under sterile conditions, a mastoidectomy was performed. Two small holes were made at the junction of the ampullae of the superior and horizontal semicircular canals, keeping a narrow strut of bone intact between the two to serve as a stop when inserting the forked electrode array. An opening was also made in the thin segment of the posterior semicircular canal near its junction with the ampulla, into which a single electrode array was inserted. Reference electrodes were inserted into the common crus of the labyrinth and in extracranial musculature. Pieces of fascia were tucked around each array, and bone chips or a small amount of dental cement was used to stabilize the electrode leads, which were run under the periosteum to the head cap. The animals were allowed 2 weeks to recover from surgery before any experiments were performed.

### Data acquisition

During recording sessions, monkeys were comfortably seated in a primate chair, set on a vestibular turntable. An enamel-insulated tungsten microelectrode (8–10 MΩ impedance; Frederick Haer Co., Bowdoinham, ME) was used to record single unit activity of vestibular nuclei neurons from three animals (Monkeys O, J & Y). Extracellular single-unit activity of afferents innervating the horizontal semicircular canal was recorded from one animal (Monkey Y) using glass microelectrodes filled with 3 M NaCl, 20–25  MΩ (ref. 56). Horizontal and vertical gaze and head position (recorded using the magnetic search coil technique), turntable velocity (measured using an angular velocity sensor (Watson Inc.)), and target position were low-pass filtered (250 Hz, analogue 8 pole Bessel filter), and sampled at 1 kHz. Unit activity was sampled at 40 kHz. An adaptive filter (Artifact Zapper, Riverbend Instruments) was used on-line to remove artefacts in the neural recordings caused by stimulation of the vestibular nerve^57^. If any residual artefact remained, we performed a template deletion off-line, where we computed an average waveform of the artefact and subtracted it from the recording trace aligned at the onset of each stimulus pulse^58^. All signals were saved using a computer-based data acquisition system (Plexon). Each unit was analysed off-line to ensure proper isolation. Subsequent analysis was performed using custom algorithms (Matlab, The MathWorks).

### Experimental paradigms

The location of the vestibular nucleus was determined relative to that of the abducens nucleus, identified by its stereotypical discharge pattern during eye movements^15^,^54^. Neurons were initially recorded during standard head-restrained paradigms to characterize their sensitivities to eye movements and head velocity. Saccadic eye movements were elicited by stepping a laser target that the animal was trained to follow between horizontal positions (±5, 10, 15, 20, 25 and 30°). Smooth pursuit eye movements were evoked by rotating a target sinusoidally in the horizontal plane with a frequency of 0.5 Hz (±40° per second). Neuronal sensitivities to head velocity were quantified by passively rotating monkeys about an earth-vertical axis (with a frequency of 0.5 Hz, ±40° per second) in the dark. The vestibular nerve was approached through the floccular complex, which was identified by its eye movement-related activity^56^.

After the basic characterization of vestibular nuclei neurons and afferent fibres, we recorded neuronal activity in complete darkness while monkeys were head-fixed during the delivery of electrical pulses to electrodes implanted in the horizontal semicircular canal. Pulses were applied using an isolated pulse stimulator (A-M systems), which was set to deliver symmetric biphasic pulses (150 μs per phase; Fig. 1b). Maximum current amplitudes were set to 80% of the minimum value at which facial muscle activation was visible using biphasic pulses delivered at 300 p.p.s. Each experimental trial consisted of three ‘activation blocks' during which we consistently activated the vestibular nerve by applying tetanic stimulation: 30 pulse trains each lasting 500 ms were delivered every 2 s over the course of 1 min at pulse rates of 100, 200 and 300 p.p.s. (Fig. 1b). Before and after each activation block, we delivered 1 s pulse trains with rates ranging from 25 to 300 p.p.s. to the vestibular nerve (that is, test blocks). We chose this range of pulse rates because they fall within the physiological range of vestibular afferent firing rates^56^. These test blocks served to quantify eye movement and neuronal sensitivities to stimulation with a vestibular prosthesis before and after the activation blocks. We also applied our test blocks every 2 min for up to 10 min following vestibular nerve activation with 300 p.p.s.

### Analysis of neuronal discharges

Gaze, head, target and turntable signals were digitally filtered at 125 Hz. Eye position was calculated from the difference between gaze and head position signals. Gaze, eye and head position signals were digitally differentiated to produce velocity signals. Neuronal firing rates were estimated using a Gaussian window (s.d. of 10 ms)^59^.

In this study, we present data from two classes of neurons in the medial vestibular nuclei that were sensitive to yaw rotations: (i) PVP neurons, which encode eye position during fixation, respond to smooth pursuit eye movement, and pause during saccades, and (ii) EH neurons, which are sensitive to head velocity during cancellation of the VOR and eye velocity in the same direction during smooth pursuit^2^. To distinguish between these two types of neurons, periods of steady fixation and saccade-free smooth pursuit were analysed using a multiple regression analysis and correlations between firing rate and eye position/velocity were assessed. A least squares regression analysis was then used to analyse the responses of both classes of neurons during passive head rotations. To quantify response modulation during whole-body rotation, we optimized the coefficients of the following equation:


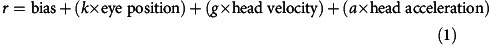


where *r* is the firing rate, bias is a constant equal to the resting neural discharge, *k* is the cell's sensitivity to eye position, and *g* and *a* are constant coefficients. The estimates of *g* and *a* were then used to calculate the neuron's sensitivity with respect to head velocity. Neuronal sensitivities (*S* in spikes per second per degree per second) and phases (*ϕ* in degrees) relative to head velocity were then computed using the following equations:


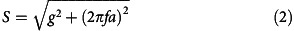



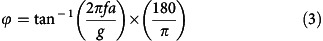


where *f* is the frequency of the sinusoidal rotation.

To quantify neuronal responses and eye movements during the test blocks, standard linear regression techniques were used to describe the relationships between (i) firing rate and pulse rate and (ii) horizontal eye velocity and pulse rate. Only periods of slow-phase eye movements were included in these analyses. Then to quantify the precise time course of neuronal responses to vestibular nerve activation, we employed a method similar to that described by Broussard and Lisberger^60^. Specifically, we measured the latency at which the first spike occurred after each pulse (delivered at 25 p.p.s.) and calculated the probability of a spike occurring as a function of latency from pulse onset. We approximated the contribution of spontaneous firing by overlaying a ‘fake' pulse train (25 p.p.s.) on a cell's baseline activity and calculating the spontaneous probability of a spike occurring as a function of latency from pulse onset. We derived the change in probability of firing from baseline by subtracting the spontaneous probability of firing from the raw probability of firing (shown in Figs 2h and 5f–i). The magnitude of the response was calculated as the difference between the maximum response after stimulation and baseline activity. Latency was defined as the *x* intercept of the linear regression of the rise or decrease in the net probability of firing. In addition, the regularity of the resting discharge of each neuron was measured by computing its coefficient of variation (CV), 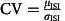, where *μ*_ISI_ and *σ*_ISI_ are the mean and s.d. of the interspike intervals (ISIs). Note, this analysis was limited to the segments of data collected when the animal was fixating at positions of <5° relative to midline.

Throughout the Results, values are expressed as mean±s.e.m. and a Student's *t*-test was used to determine whether the average of two measured parameters differed significantly from each other.

## Additional information

**How to cite this article:** Mitchell, D. E. *et al*. Plasticity within non-cerebellar pathways rapidly shapes motor performance *in vivo*. *Nat. Commun.* 7:11238 doi: 10.1038/ncomms11238 (2016).

## Supplementary Material

Supplementary Information Supplementary Figures 1-4

## References

[b1] de NÓ R. L. Vestibulo-ocular reflex arc. Arch. Neurol. Psychiatry 30, 245 (1933).

[b2] Cullen K. E. The vestibular system: multimodal integration and encoding of self-motion for motor control. Trends Neurosci. 35, 185–196 (2012). 22245372 10.1016/j.tins.2011.12.001 PMC4000483

[b3] Boyden E. S., Katoh A. & Raymond J. L. Cerebellum-dependent learning: the role of multiple plasticity mechanisms. Annu. Rev. Neurosci. 27, 581–609 (2004). 15217344 10.1146/annurev.neuro.27.070203.144238

[b4] Lisberger S. G. Internal models of eye movement in the floccular complex of the monkey cerebellum. Neuroscience 162, 763–776 (2009). 19336251 10.1016/j.neuroscience.2009.03.059 PMC2740815

[b5] Allum J. H., Yamane M. & Pfaltz C. R. Long-term modifications of vertical and horizontal vestibulo-ocular reflex dynamics in man. I. After acute unilateral peripheral vestibular paralysis. Acta Otolaryngol. 105, 328–337 (1988). 3389119 10.3109/00016488809097015

[b6] Curthoys I. S. & Halmagyi G. M. Vestibular compensation: a review of the oculomotor, neural, and clinical consequences of unilateral vestibular loss. J. Vestib. Res. 5, 67–107 (1995). 7743004

[b7] Sadeghi S. G., Minor L. B. & Cullen K. E. Dynamics of the horizontal vestibuloocular reflex after unilateral labyrinthectomy: response to high frequency, high acceleration, and high velocity rotations. Exp. Brain Res. 175, 471–484 (2006). 16957885 10.1007/s00221-006-0567-7

[b8] Broussard D. M., Titley H. K., Antflick J. & Hampson D. R. Motor learning in the VOR: the cerebellar component. Exp. Brain Res. 210, 451–463 (2011). 21336828 10.1007/s00221-011-2589-z

[b9] Miles F. A. & Lisberger S. G. Plasticity in the vestibulo-ocular reflex: a new hypothesis. Annu. Rev. Neurosci. 4, 273–299 (1981). 6784658 10.1146/annurev.ne.04.030181.001421

[b10] Gittis A. H. & du Lac S. Intrinsic and synaptic plasticity in the vestibular system. Curr. Opin. Neurobiol. 16, 385–390 (2006). 16842990 10.1016/j.conb.2006.06.012

[b11] McElvain L. E., Bagnall M. W., Sakatos A. & du Lac S. Bidirectional plasticity gated by hyperpolarization controls the gain of postsynaptic firing responses at central vestibular nerve synapses. Neuron 68, 763–775 (2010). 21092864 10.1016/j.neuron.2010.09.025 PMC3189222

[b12] Scarduzio M., Panichi R., Pettorossi V. E. & Grassi S. The repetition timing of high frequency afferent stimulation drives the bidirectional plasticity at central synapses in the rat medial vestibular nuclei. Neuroscience 223, 1–11 (2012). 22863673 10.1016/j.neuroscience.2012.07.039

[b13] Cullen K. E. & Roy J. E. Signal processing in the vestibular system during active versus passive head movements. J. Neurophysiol. 91, 1919–1933 (2004). 15069088 10.1152/jn.00988.2003

[b14] Scudder C. A. & Fuchs A. F. Physiological and behavioral identification of vestibular nucleus neurons mediating the horizontal vestibuloocular reflex in trained rhesus monkeys. J. Neurophysiol. 68, 244–264 (1992). 1517823 10.1152/jn.1992.68.1.244

[b15] Cullen K. E. & McCrea R. A. Firing behavior of brain stem neurons during voluntary cancellation of the horizontal vestibuloocular reflex. I. Secondary vestibular neurons. J. Neurophysiol. 70, 828–843 (1993). 8410175 10.1152/jn.1993.70.2.828

[b16] Goldberg J. M., Smith C. E. & Fernández C. Relation between discharge regularity and responses to externally applied galvanic currents in vestibular nerve afferents of the squirrel monkey. J. Neurophysiol. 51, 1236–1256 (1984). 6737029 10.1152/jn.1984.51.6.1236

[b17] Aw S. T., Todd M. J., Aw G. E., Weber K. P. & Halmagyi G. M. Gentamicin vestibulotoxicity impairs human electrically evoked vestibulo-ocular reflex. Neurology 71, 1776–1782 (2008). 19029517 10.1212/01.wnl.0000335971.43443.d9

[b18] Malinvaud D., Vassias I., Reichenberger I., Rössert C. & Straka H. Functional organization of vestibular commissural connections in frog. J. Neurosci. 30, 3310–3325 (2010). 20203191 10.1523/JNEUROSCI.5318-09.2010 PMC6634120

[b19] Graham B. P. & Dutia M. B. Cellular basis of vestibular compensation: analysis and modelling of the role of the commissural inhibitory system. Exp. Brain Res. 137, 387–396 (2001). 11355384 10.1007/s002210100677

[b20] Shimazu H. & Precht W. Inhibition of central vestibular neurons from the contralateral labyrinth and its mediating pathway. J. Neurophysiol. 29, 467–492 (1966). 5961161 10.1152/jn.1966.29.3.467

[b21] Shimazu H. Organization of the commissural connections: physiology. Prog. Brain Res. 37, 177–190 (1972). 4345122 10.1016/S0079-6123(08)63902-3

[b22] Markham C. H., Yagi T. & Curthoys I. S. The contribution of the contralateral labyrinth to second order vestibular neuronal activity in the cat. Brain Res. 138, 99–109 (1977). 589471 10.1016/0006-8993(77)90786-7

[b23] Dieringer N. & Precht W. Mechanisms of compensation for vestibular deficits in the frog. I. Modification of the excitatory commissural system. Exp. Brain Res. 36, 311–328 (1979). 226388 10.1007/BF00238914

[b24] Dieringer N. & Precht W. Mechanisms of compensation for vestibular deficits in the frog. II. Modification of the inhibitory Pathways. Exp. Brain Res. 36, 329–357 (1979). 314903 10.1007/BF00238915

[b25] Straka H. & Dieringer N. Uncrossed disynaptic inhibition of second-order vestibular neurons and its interaction with monosynaptic excitation from vestibular nerve afferent fibers in the frog. J. Neurophysiol. 76, 3087–3101 (1996). 8930257 10.1152/jn.1996.76.5.3087

[b26] Linden D. J. Long-term synaptic depression in the mammalian brain. Neuron 12, 457–472 (1994). 8155315 10.1016/0896-6273(94)90205-4

[b27] Linden D. J. & Connor J. A. Long-term synaptic depression. Annu. Rev. Neurosci. 18, 319–357 (1995). 7605065 10.1146/annurev.ne.18.030195.001535

[b28] Grassi S. & Pettorossi V. E. Synaptic plasticity in the medial vestibular nuclei: role of glutamate receptors and retrograde messengers in rat brainstem slices. Prog. Neurobiol. 64, 527–553 (2001). 11311461 10.1016/s0301-0082(00)00070-8

[b29] Murashima M. & Hirano T. Entire course and distinct phases of day-lasting depression of miniature EPSC amplitudes in cultured Purkinje neurons. J. Neurosci. 19, 7326–7333 (1999). 10460239 10.1523/JNEUROSCI.19-17-07326.1999 PMC6782505

[b30] Sadeghi S. G., Minor L. B. & Cullen K. E. Neural correlates of motor learning in the vestibulo-ocular reflex: dynamic regulation of multimodal integration in the macaque vestibular system. J. Neurosci. 30, 10158–10168 (2010). 20668199 10.1523/JNEUROSCI.1368-10.2010 PMC2933842

[b31] Sadeghi S. G., Minor L. B. & Cullen K. E. Neural correlates of sensory substitution in vestibular pathways following complete vestibular loss. J. Neurosci. 32, 14685–14695 (2012). 23077054 10.1523/JNEUROSCI.2493-12.2012 PMC3503523

[b32] Kotak V. C. . Hearing loss raises excitability in the auditory cortex. J. Neurosci. 25, 3908–3918 (2005). 15829643 10.1523/JNEUROSCI.5169-04.2005 PMC1764814

[b33] Maffei A. & Turrigiano G. G. Multiple modes of network homeostasis in visual cortical layer 2/3. J. Neurosci. 28, 4377–4384 (2008). 18434516 10.1523/JNEUROSCI.5298-07.2008 PMC2655203

[b34] Dale A., Carriot J. & Cullen K. in *Neuroscience Meeting Planner*. Programme No. 265.09 (San Diego, CA, Society for Neuroscience, 2013).

[b35] Yu X.-J., Thomassen J. S., Dickman J. D., Newlands S. D. & Angelaki D. E. Long-term deficits in motion detection thresholds and spike count variability after unilateral vestibular lesion. J. Neurophysiol. 112, 870–889 (2014). 24848470 10.1152/jn.00280.2014 PMC4122753

[b36] Goel A. & Buonomano D. V. Chronic electrical stimulation homeostatically decreases spontaneous activity, but paradoxically increases evoked network activity. J. Neurophysiol. 109, 1824–1836 (2013). 23324317 10.1152/jn.00612.2012 PMC3628006

[b37] Ouardouz M. & Sastry B. R. Mechanisms underlying LTP of inhibitory synaptic transmission in the deep cerebellar nuclei. J. Neurophysiol. 84, 1414–1421 (2000). 10980014 10.1152/jn.2000.84.3.1414

[b38] Shin M. . Multiple types of cerebellar target neurons and their circuitry in the vestibulo-ocular reflex. J. Neurosci. 31, 10776–10786 (2011). 21795530 10.1523/JNEUROSCI.0768-11.2011 PMC3227528

[b39] Koekkoek S. K. E. . Cerebellar LTD and learning-dependent timing of conditioned eyelid responses. Science 301, 1736–1739 (2003). 14500987 10.1126/science.1088383

[b40] De Zeeuw C. I. . Expression of a protein kinase C inhibitor in Purkinje cells blocks cerebellar LTD and adaptation of the vestibulo-ocular reflex. Neuron 20, 495–508 (1998). 9539124 10.1016/s0896-6273(00)80990-3

[b41] Koekkoek S. K. E. . Deletion of FMR1 in Purkinje cells enhances parallel fiber LTD, enlarges spines, and attenuates cerebellar eyelid conditioning in Fragile X syndrome. Neuron 47, 339–352 (2005). 16055059 10.1016/j.neuron.2005.07.005

[b42] Ke M. C., Guo C. C. & Raymond J. L. Elimination of climbing fiber instructive signals during motor learning. Nat. Neurosci. 12, 1171–1179 (2009). 19684593 10.1038/nn.2366 PMC3864777

[b43] Nguyen-Vu T. D. B. . Cerebellar Purkinje cell activity drives motor learning. Nat. Neurosci. 16, 1734–1736 (2013). 24162651 10.1038/nn.3576 PMC3966616

[b44] Schonewille M. . Reevaluating the role of LTD in cerebellar motor learning. Neuron 70, 43–50 (2011). 21482355 10.1016/j.neuron.2011.02.044 PMC3104468

[b45] Boyden E. S. . Selective engagement of plasticity mechanisms for motor memory storage. Neuron 51, 823–834 (2006). 16982426 10.1016/j.neuron.2006.08.026

[b46] Welsh J. P. . Normal motor learning during pharmacological prevention of Purkinje cell long-term depression. Proc. Natl Acad. Sci. USA 102, 17166–17171 (2005). 16278298 10.1073/pnas.0508191102 PMC1288000

[b47] Beraneck M., McKee J. L., Aleisa M. & Cullen K. E. Asymmetric recovery in cerebellar-deficient mice following unilateral labyrinthectomy. J. Neurophysiol. 100, 945–958 (2008). 18509072 10.1152/jn.90319.2008 PMC2525728

[b48] van de Berg R. . The vestibular implant: frequency-dependency of the electrically evoked vestibulo-ocular reflex in humans. Front. Syst. Neurosci. 8, 255 (2014). 25653601 10.3389/fnsys.2014.00255 PMC4299437

[b49] Phillips J. O. . Vestibular implantation and longitudinal electrical stimulation of the semicircular canal afferents in human subjects. J. Neurophysiol. 113, 3866–3892 (2015). 25652917 10.1152/jn.00171.2013 PMC4480623

[b50] Mitchell D. E. . Head movements evoked in alert rhesus monkey by vestibular prosthesis stimulation: implications for postural and gaze stabilization. PLoS ONE 8, e78767 (2013). 24147142 10.1371/journal.pone.0078767 PMC3798420

[b51] Dai C. . Directional plasticity rapidly improves 3D vestibulo-ocular reflex alignment in monkeys using a multichannel vestibular prosthesis. J. Assoc. Res. Otolaryngol. 14, 863–877 (2013). 24013822 10.1007/s10162-013-0413-0 PMC3825024

[b52] Merfeld D. M. & Lewis R. F. Replacing semicircular canal function with a vestibular implant. Curr. Opin. Otolaryngol. Head Neck Surg. 20, 386–392 (2012). 22886037 10.1097/MOO.0b013e328357630f

[b53] Dadarlat M. C., O'Doherty J. E. & Sabes P. N. A learning-based approach to artificial sensory feedback leads to optimal integration. Nat. Neurosci. 18, 138–144 (2014). 25420067 10.1038/nn.3883 PMC4282864

[b54] Sylvestre P. A. & Cullen K. E. Quantitative analysis of abducens neuron discharge dynamics during saccadic and slow eye movements. J. Neurophysiol. 82, 2612–2632 (1999). 10561431 10.1152/jn.1999.82.5.2612

[b55] Dai C. . Restoration of 3D vestibular sensation in rhesus monkeys using a multichannel vestibular prosthesis. Hear Res. 281, 74–83 (2011). 21888961 10.1016/j.heares.2011.08.008 PMC3254699

[b56] Sadeghi S. G., Minor L. B. & Cullen K. E. Response of vestibular-nerve afferents to active and passive rotations under normal conditions and after unilateral labyrinthectomy. J. Neurophysiol. 97, 1503–1514 (2007). 17122313 10.1152/jn.00829.2006

[b57] Gnadt J. W., Echols S. D., Yildirim A., Zhang H. & Paul K. Spectral cancellation of microstimulation artifact for simultaneous neural recording in situ. IEEE Trans. Biomed. Eng. 50, 1129–1135 (2003). 14560765 10.1109/TBME.2003.816077

[b58] Brontë-Stewart H. M. & Lisberger S. G. Physiological properties of vestibular primary afferents that mediate motor learning and normal performance of the vestibulo-ocular reflex in monkeys. J. Neurosci. 14, 1290–1308 (1994). 8120625 10.1523/JNEUROSCI.14-03-01290.1994 PMC6577573

[b59] Cullen K. E., Rey C. G., Guitton D. & Galiana H. L. The use of system identification techniques in the analysis of oculomotor burst neuron spike train dynamics. J. Comput. Neurosci. 3, 347–368 (1996). 9001977 10.1007/BF00161093

[b60] Broussard D. M. & Lisberger S. G. Vestibular inputs to brain stem neurons that participate in motor learning in the primate vestibuloocular reflex. J. Neurophysiol. 68, 1906–1909 (1992). 1479453 10.1152/jn.1992.68.5.1906

