# Supplementary material for: Plasticity within non-cerebellar pathways rapidly shapes motor performance in vivo
Source: Nat Commun. 2016 May 9;7:11238. doi: 10.1038/ncomms11238 (PMC4865756; doi:10.1038/ncomms11238)
Supplement: Supplementary Information — Supplementary Figures 1-4 [file ncomms11238-s1.pdf]

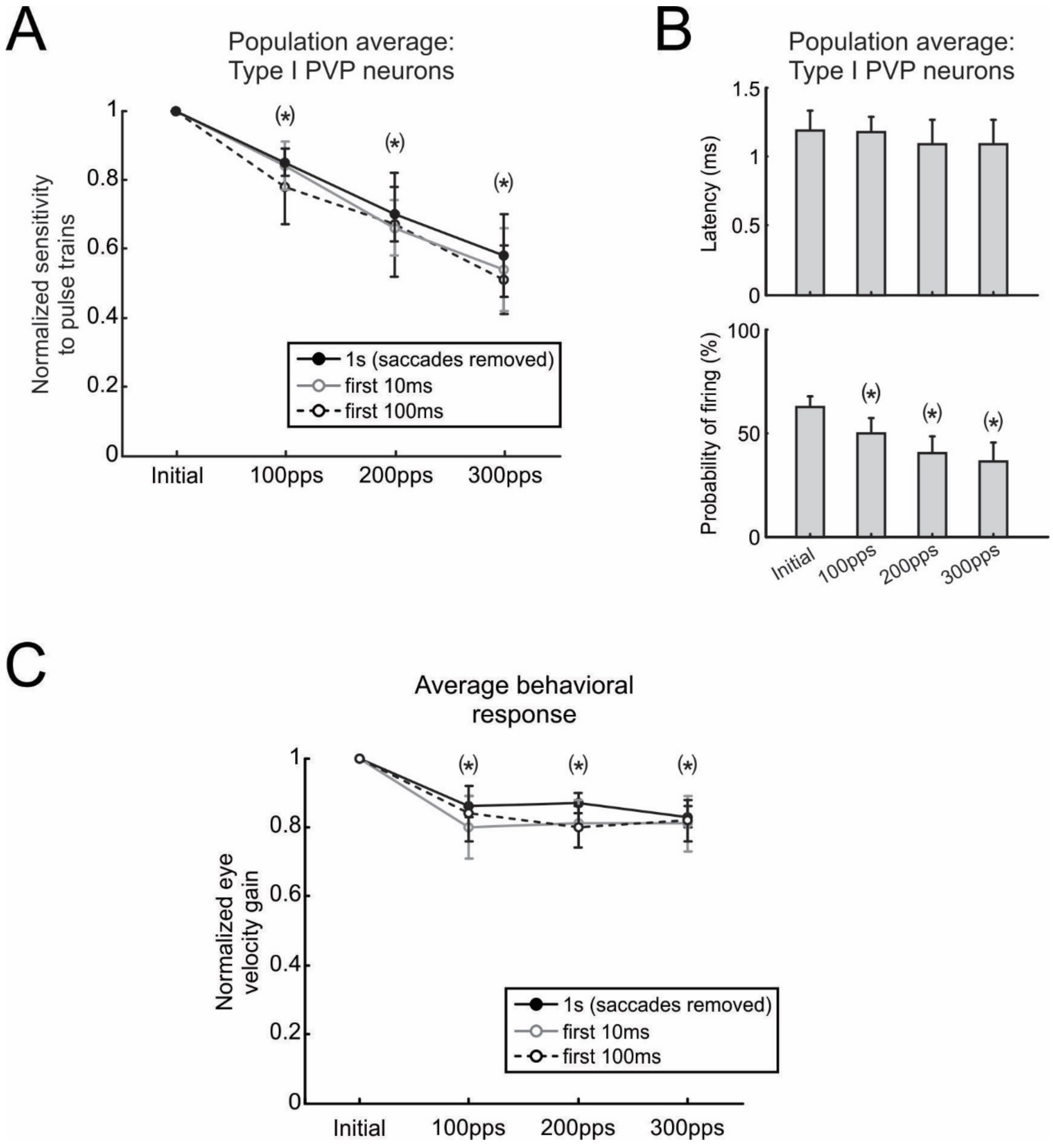


**Supplementary Figure 1:** Type I PVP and behavioral responses remain attenuated across different timescales. *(A)* Comparison of type I PVP responses calculated during compensatory portion of the eye movement during entire 1s pulse trains, as well as the first 10ms and 100ms following the onset of the pulse train (n=15). *P<0.05 (Student's t-test). ***(B)*** Upper panel: Latency calculated based on the onset of probability of firing curves. Lower panel: Magnitude of probability of firing response. ***(C)*** Comparison of behavioral responses calculated during compensatory portion of the eye movement during entire 1s pulse trains, as well as the first 10ms and 100ms following the onset of the pulse train (n=61). *P<0.05 (Student's t-test).

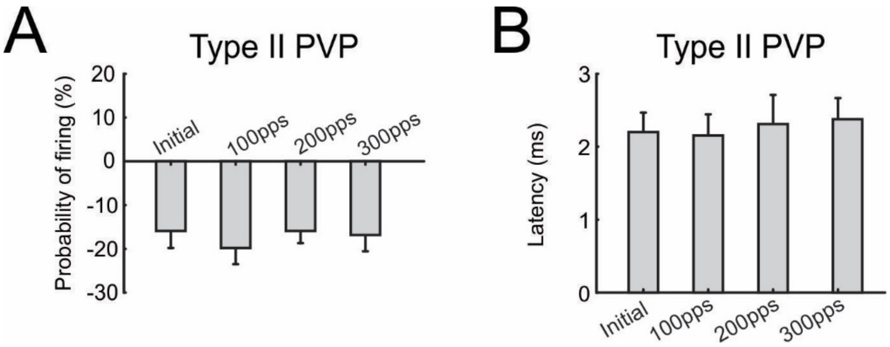


**Supplementary Figure 2:** Magnitude and latency of response to individual pulses remain constant for type II PVP neurons. ***(A)*** Magnitude of probability of firing response. ***(B)*** Latency calculated based on the onset of probability of firing curves (n=10).

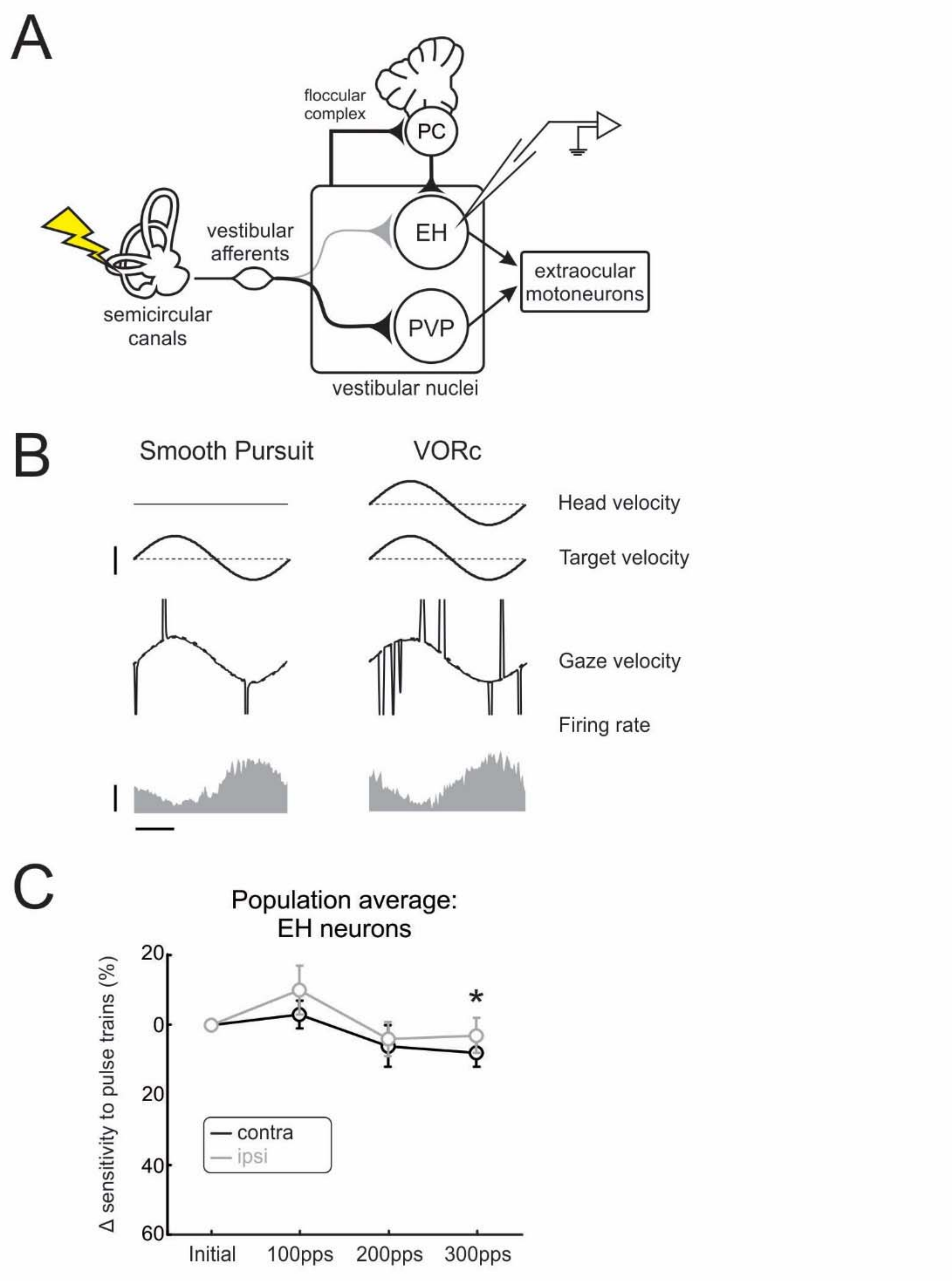


**Supplementary Figure 3:** Weight of cerebellar VOR pathway remains constant following activation. ***(A)*** Diagram of cerebellar VOR pathways. ***(B)*** Head, target and gaze velocity as well as example contra EH cell firing rate during smooth pursuit eye movements (left panels) and when the monkey canceled its VOR by tracking a target that moved with the same velocity as its head (right panels). Calibrations bars: 40° per s, 100 spikes per s, 0.5s. ***(C)*** Population average of normalized sensitivity to test stimulation for contra (n=14) and ipsi (n=12) EH cells before and after activation of the vestibular nerve. *P<0.05 (Student's t-test).

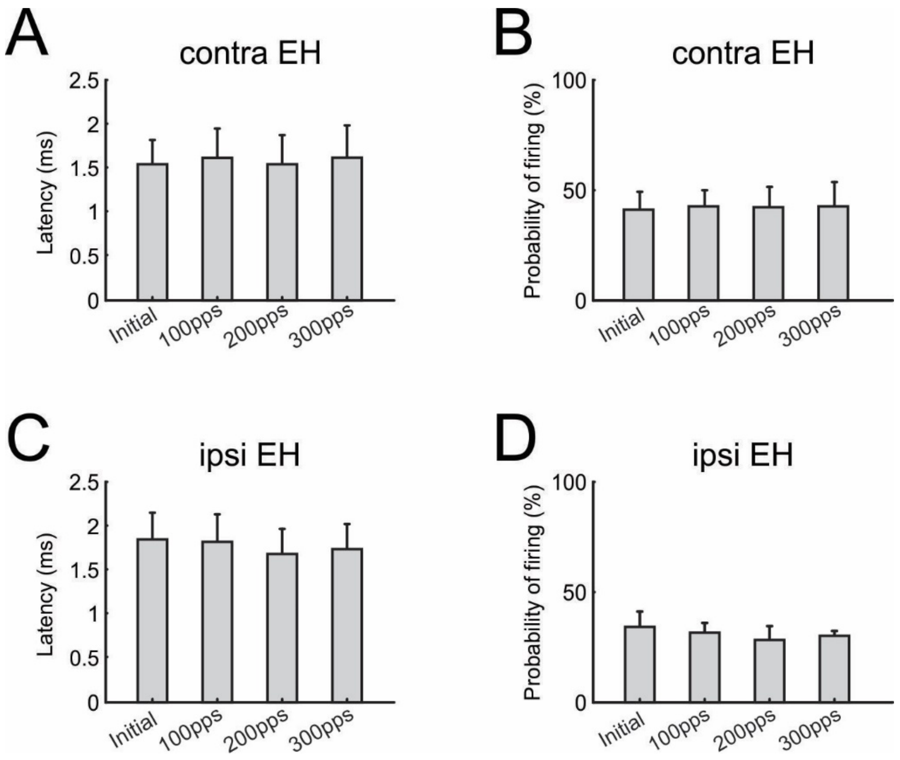


**Supplementary Figure 4:** Latency and magnitude of response to individual pulses remain constant for EH cells. ***(A, C)*** Latency calculated based on the onset of probability of firing curves. ***(B, D)*** Magnitude of probability of firing response (n=14 and 12, for contra and ipsi EH cells, respectively).
